# Effectiveness of the Homeopathic Preparation Neurexan^®^ Compared with that of Commonly used Valerian-Based Preparations for the Treatment of Nervousness/Restlessness – an Observational Study

**DOI:** 10.1100/tsw.2009.95

**Published:** 2009-08-11

**Authors:** Rainer Hubner, Robbert van Haselen, Peter Klein

**Affiliations:** Westring 20, Landau, Germany; ^2^ Biologische Heilmittel Heel GmbH, Baden-Baden, Germany; ^3^ D.s.h. Statistical Services GmbH, Rohrbach, Germany

**Keywords:** complementary medicine, homeopathy, tolerance, stress, homotoxicology

## Abstract

Mild anxieties, nervousness, and restlessness are common in the general population and are commonly treated by complementary and alternative medical (CAM) therapies. A prospective, nonrandomized, noninterventional, observational study, using conventional or CAM practices, was conducted in 49 German practices. Each practice could include up to 15 subjects treated with either the homeopathic preparation Neurexan^®^ or with combination formulations based on valerian extracts. There was no placebo group. Choice and doses of study therapies were at the respective physician's discretion. The planned treatment duration was 2 weeks. A total of 826 subjects were included in the study and 777 (553 Neurexan and 224 valerian) subjects were available for the final examination. Subjects receiving Neurexan tended to weigh less, to have fewer concomitant illnesses and slightly milder severity of nervousness/restlessness, and were likelier to be female than the subjects receiving valerian therapies. The summary score for nervousness/restlessness was reduced from 19.0 ± 6.1 at baseline to 7.4 ± 6.8 at the end of the observation period in the Neurexan group, a reduction of 11.5 ± 7.3 score units. In the valerian group, the summary score was reduced from 21.4 ± 6.0 to 12.6 ± 7.3, a reduction of 9.0 ± 6.6 score units. The changes from baseline and the differences between the groups were statistically significant. Similar significant differences in effects were seen on the subscores and on the subjects' assessments of effectiveness. Both study therapies were well tolerated. Neurexan appears to be an effective and well-tolerated alternative to valerian-based combination therapies for the treatment of nervousness/restlessness in subjects favorable towards a CAM-based therapy.

### INTRODUCTION

Mild anxieties, nervousness, and restlessness are common in the general population. The range of treatments is very broad and medications range from benzodiazepines to transcendental meditation[1] and homeopathic preparations[2]. Self-medication is also common, including the use of alcohol[3]. Many conventional medications are associated with undesirable side effects, particularly with long-term use. The detrimental effects of alcohol on physical and psychological well-being are well known. The commonly prescribed benzodiazepines are contraindicated for long-term use because of their addictiveness and side effects[4].

The practice of complementary and alternative medicine (CAM) is gaining increasing popularity worldwide[5,6,7]. The symptomatic treatment of ailments of psychiatric or nervous origin is an area with relatively high use of CAM therapies. Good results have been reported with various CAM treatments for insomnia[8,9], mild nervous disorders[10], and psychological traumas[11]. A commonly cited advantage of CAM medications is their superior tolerability profile to many pharmacotherapies[12]. However, the implementation of CAM practices in daily use by greater numbers of practitioners is hampered by the insufficiently documented effectiveness of many treatments.

Two comparatively well-investigated CAM therapies with anxiolytic effects are herbal preparations based on valerian and the homeopathic therapy Neurexan® (Heel GmbH, Baden-Baden Germany). Valerian-based combination therapies are widely used as mild sedatives and anxiolytics, with a body of evidence from observational as well as randomized studies supporting their efficacy[13,14,15]. Neurexan is an over-the-counter preparation based on the principles of homotoxicology[16], which uses a combination of components at lower potencies than in classical homeopathy: $10^{-2}$ to $10^{-4}$ rather than the $10^{-8}$ dilutions typical in homeopathy. In a recent observational study, Neurexan was shown to be an effective and well-tolerated alternative to valerian-based therapies for the treatment of mild to moderate insomnia[8].

To gather more data on the effectiveness of Neurexan in settings of routine CAM practice, we conducted a large-scale, prospective, observational study on the effectiveness of Neurexan (tablets) in the treatment of nervousness/restlessness. The effectiveness was compared with that of common combination formulations based on valerian extracts. The study design was chosen in order to capture the variety of subjects treated in everyday CAM practice.

### METHODS

#### Study Design

This was a prospective, two-arm, nonrandomized, noninterventional, observational study, conducted in 49 German general practices between 3 April 2006 (first subject included) and 27 December 2006 (last subject completed).

Subjects had clinical symptoms of nervousness/restlessness, either as judged by the evaluating clinician or defined as the presence of one or several of the following signs and symptoms: excitability/jitteriness, hyperactivity; sleep disturbances; fitful sleep or significant phases of sleeplessness; nocturnal anxiety; difficulties with concentration/forgetfulness; fatigue; listlessness; moroseness; gastrointestinal disturbances; or headache/pressure. Subjects were excluded if currently receiving medication for nervous restlessness, psychopharmacotherapy or homeopathic drugs with psychological effects, and if there were signs of alcohol/drug abuse or psychiatric disorders. It was projected that each practice could include up to 15 subjects treated with either Neurexan or with combination formulations based on valerian extracts.

### Treatments

Included practices could specialize in CAM or in conventional medicine. Treatment and dosage was at the discretion of the physician. Subjects who received either Neurexan tablets or valerian-based combination preparations in clinical routine could be observed and documented. Neurexan is based on highly diluted plant extracts, including valerianate of zinc (Table 1)[17]. All components are listed in the German Homeopathic Pharmacopoeia[18]. The recommended daily dose of Neurexan is 3×1 tablets. In acute cases, single tablets every 30–60 min are recommended for temporary symptomatic relief, up to a maximum of 12 tablets daily. Likewise, for valerian-based therapy, physicians had full freedom to choose from available commercial preparations. Medications for nervous restlessness other than the study medications, psychopharmacotherapy, or homeopathic drugs with psychological effects were not allowed for the duration of the study. Concomitant medications were documented.

**TABLE 1**
**Components of Neurexan and Their Dilutions**

| Component | Common Name | Dilution | mg in each tablet |
|---|---|---|---|
| *Passiflora incarnata* | White sarsaparilla | D2 | 0.6 |
| *Avena sativa* | Common oats | D2 | 0.6 |
| *Coffea arabica* | Coffee tree | D12 | 0.6 |
| *Zincum isovalerianicum* | Valerianate of zinc | D4 | 0.6 |

The study was performed in accordance with the recommendations for the conduct of observational studies by the German Federal Institute for Medicine and Medicinal Products[19] in full compliance with the principles of the Declaration of Helsinki[20].

### Analyses

The planned duration of observation was 2 weeks. Subjects were examined at the time of entering the study, optionally after 1 week's treatment, and at a final visit at the end of the study. At the entry visit, demographic data (sex, ethnic origin, age, body height, body weight, body mass index) were collected, as well as data on vital statistics, degree of symptoms, previous therapy, duration of disease, etiology, severity of symptoms, physician-evaluated overall severity of disease, any clinically relevant concomitant diseases, additional medical and nonmedical treatments of underlying and concomitant diseases, and the impact of the illness on subjects' professional life and ability to work. At the final visit, and at the optional interim visit, records were made of adverse drug reactions, changes in dosages of study medication and/or other medications, severity of symptoms, overall severity of disease, and the impact of the disease on subjects' professional lives.

The effectiveness analysis was carried out on data from the final examination. Treatment effectiveness was graded by the treating physician in a dialogue with the subjects. The effectiveness variables were the individual signs and symptoms included in the diagnosis of nervousness/restlessness, as well as the sum of the individual symptom scores. Severity of symptoms was graded on a four-point scale from 0 to 3, where 0 indicated asymptomatic, 1 mild, 2 moderate, and 3 severe symptoms. The change from baseline to the end of the study period was calculated for individual symptomatic scores and for the summary score (0–36) of the individual symptom scores.

Further, the improvement vs. baseline in the subjects' perceived global state of nervousness/restlessness was assessed at the end of the study on a scale from very much improved, much improved, slightly improved, no change, slightly worsened, to very much worsened. Also evaluated were changes in the subjects' overall health status from baseline to the end of the treatment period. This evaluation was carried out by the physician based on subject feedback with the status graded as excellent, good, satisfactory, no improvement, or worsened. Subjects also recorded their first experience of symptomatic improvement with therapy (within 24 h, after 2 days, after 3 days, after 4–7 days, after 8–14 days, after >14 days, no improvement).

Tolerability data were gathered as adverse events. In addition, the overall tolerability of the treatment regimens were assessed by the physician in dialogue with the subject as excellent (no symptoms of intolerance), good (occasional symptoms of intolerance), moderate (frequent occurrence of such symptoms), or poor (symptoms of intolerance following each administration of study drug). Compliance was rated by the physician at the final visit on a rating scale from excellent (subjects adhered strictly to the treatment scheme), good (subjects adhered largely to the treatment scheme), moderate (spurious adherence), to poor (no adherence).

### Statistical Methods

All analyses were exploratory. Summary statistics were calculated using absolute symptom scores and percentages of patients for baseline characteristics. Differences in baseline characteristics between treatment groups were adjusted by propensity scores[21,22] using the baseline characteristics demographic data, course/type of disease, cause of disease, degree of disease severity, and concomitant diseases as covariates. On the basis of these baseline factors, for each patient, the propensity score was estimated using logistic regression ($0 \leq$ propensity score $\leq 1$). The classification was done in five, approximately equal, propensity-score classes. Between-groups differences were evaluated by ANOVA (interval scaled data), the Cochran-Mantel-Haenszel test (ordinal scaled data), and Fishers's exact test (nominal data), as appropriate. For all comparisons, two-sided 95% confidence limits were calculated.

For the efficacy analysis, the efficacy population (Fig. 1) was used. As this was an exploratory pilot study, there was no prespecified null hypothesis and all conclusions were of an exploratory nature. It was assumed that the symptomatic effectiveness of treatment with Neurexan on the variables studied would be no less effective than or superior to (i.e., noninferior to) that of common valerian therapy. Differences between the groups in the changes from baseline in all study variables, and for the summary score of all variables, were calculated on the basis of a covariance analysis with baseline values and propensity score as covariates. The statistical tests were two sided and performed at the 5% significance level. Adjustments for multiple testing were not done. A situation where the lower border of the 95% confidence interval did not cross the line of unity was considered indicative of a greater effect of the Neurexan therapy than of the valerian-based preparations. All statistical analyses were performed with SAS version 9.1.

In case of missing data on symptoms or on severity of disease, the last obtained measurement was substituted for the missing one (last observation carried forward). For the global assessment of efficacy at the end of the observation period, data were substituted according to a "worst case" scenario: for symptomatic improvement "no improvement", for overall health status "worsened", for change in overall condition "very much worsened", and for compliance "poor". For other assessments, missing values were not replaced.

## RESULTS

### Subject Population

The study was conducted in 49 practices with an average of 16 subjects treated in each center. In 27 practices, subjects were treated exclusively with Neurexan; one practice administered only valerian compounds. A total of 826 subjects were included in the study. Of these, 807 subjects (577 in the Neurexan

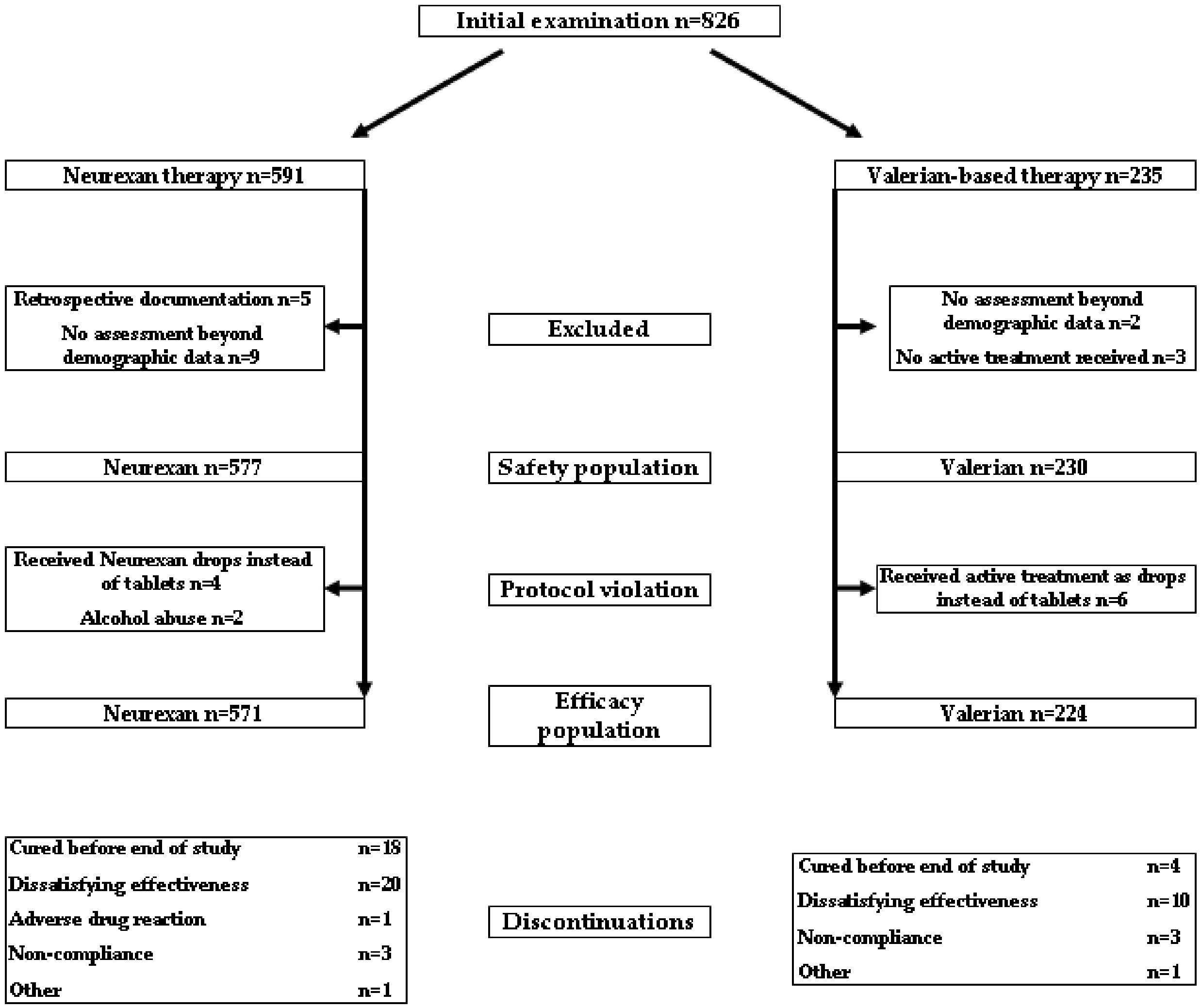

**FIGURE 1.** Subjects' disposition.

group and 230 in the valerian group) took at least one dose of study medication and were included in the safety population. Six subjects in either group were excluded as protocol violators and 795 subjects (571 receiving Neurexan, 224 valerian) were included in the efficacy population. Subject disposition is shown in Fig. 1. Forty percent of subjects in the Neurexan group and 37% of those in the valerian group attended a voluntary interim examination. A total of 553 Neurexan subjects and 224 valerian subjects were available for the final examination. The mean duration of treatment was 26 days for subjects in the Neurexan group and 21 days for subjects in the valerian group (safety and efficacy populations).

Baseline characteristics are summarized in Table 2. The treatment groups were generally well balanced. Subjects receiving Neurexan tended to weigh less, to have fewer concomitant illnesses and slightly milder severity of nervousness/restlessness, and were likelier to be female than subjects receiving valerian therapies. The overall score and the scores for fitful sleep, fatigue, and listlessness were slightly, but significantly, greater in the valerian group.

**TABLE 2**
**Baseline Characteristics**

| Parameter | Neurexan (n = 571) | Valerian (n = 224) | *p* Value for Differences Before and After Propensity Score Adjustment* |
|---|---|---|---|
| Mean age (years ± SD) | 48.2 ± 18.2 | 47.4 ± 17.7 | n.s.** |
| Women (%) | 74.3 | 67.0 | 0.0431/n.s. |
| Caucasian (%) | 98.9 | 96.4 | n.s. |
| Weight (kg ± SD) | 68.3 ± 13.9 | 71.0 ± 16.1 | 0.0182/n.s. |
| Body mass index (kg/m$^2$ mean ± SD) | 24.3 ± 4.1 | 24.5 ± 4.7 | n.s. |
| Systolic blood pressure (mmHg mean ± SD) | 125.4 ± 14.6 | 129.2 ± 15.7 | n.s. |
| Diastolic blood pressure (mmHg mean ± SD) | 76.5 ± 9.4 | 77.0 ± 10.5 | n.s. |
| Type of disease: recurrent (%) | 29.8 | 21.0 | 0.0131/n.s. |
| No concomitant illnesses (%) | 69.9 | 81.7 | 0.0007/n.s. |
| Duration of disease days (mean ± SD) | 51.6 ± 127.0 | 53.3 ± 124.1 | n.s. |
| Severity of disease | | | 0.0003/n.s. |
| Mild (%) | 12.6 | 7.1 | |
| Mild-to-moderate (%) | 41.9 | 32.6 | |
| Moderate (%) | 40.5 | 51.3 | |
| Severe (%) | 3.9 | 7.6 | |
| Symptomatic scores | | | |
| Summary score of symptoms | 19.0 ± 6.1 | 21.4 ± 6.0 | <0.0001/0.0079 |
| Nervousness/restlessness | 2.2 ± 0.7 | 2.4 ± 0.7 | 0.0110/n.s. |
| Excitability/jitteriness | 1.9 ± 0.8 | 2.1 ± 0.9 | 0.0075/n.s. |
| Hyperactivity | 1.9 ± 0.9 | 2.1 ± 0.8 | 0.0026/n.s. |
| Sleep disturbances | 2.1 ± 0.8 | 2.3 ± 0.8 | 0.0161/n.s. |
| Fitful sleep or significant phases of sleeplessness | 2.0 ± 0.9 | 2.2 ± 0.8 | 0.0007/0.0409 |
| Nocturnal anxiety | 1.2 ± 1.0 | 1.3 ± 1.1 | n.s. |
| Difficulty in concentration/forgetfulness | 1.5 ± 0.9 | 1.7 ± 0.9 | 0.0105/n.s. |
| Fatigue | 1.5 ± 0.8 | 1.7 ± 0.9 | 0.0005/0.0347 |
| Listlessness | 1.3 ± 0.9 | 1.6 ± 1.0 | <0.0001/0.0226 |
| Moroseness | 1.5 ± 0.9 | 1.7 ± 1.0 | <0.0001/n.s. |
| Gastrointestinal disturbances | 0.9 ± 0.9 | 1.1 ± 1.0 | 0.0036/n.s. |
| Headache/pressure | 1.1 ± 0.9 | 1.2 ± 1.0 | 0.0496/n.s. |
| Overall severity | 2.4 ± 0.8 | 2.6 ± 0.8 | 0.0003/n.s. |
| On sick leave (%) | 56.2 | 54.5 | n.s. |

* Adjusted for type of disease (acute, chronic, recurrent), cause and severity of disease, concomitant disease.

** n.s. = not significant.

### Study Therapies

In the Neurexan group, 283 subjects (49.6%) received a daily dose of 3×1 tablets and 245 (42.9%) received >3 tablets a day. Only 34 subjects (6.0%) received <3 tablets a day. In the valerian group, a wide range of commercially available preparations were used, all based on valerian extracts. The most commonly used combination preparation was Euvegal® (Dr. Willmar Schwabe, GmbH&Co KG,

Karlsruhe, Germany), which contains valerian extract and *Melissa* (lemon balm). This was used by 72 subjects (32.1%) in the valerian group. A similar preparation, Valeriana Hevert (Hevert GmbH & KG Nussbaum, Germany), combining valerian, lemon balm, and *Passiflora* (Passion flower), was used by 31 subjects (14%). In all, >50% of the subjects received valerian in combination with lemon balm. Other common herbal extracts used in the valerian combination formulations were *Hypericum* (St. John's Wort) and *Humulus* (Hops), in varying combinations. However, the range of valerian-based combination therapies was very wide and no single preparation other than the two mentioned above was used in >10% of the subjects.

In both groups, additional therapies for nervousness/restlessness were infrequent: physiotherapy was used by 22 Neurexan subjects (4%) and six valerian subjects (3%), respectively, and 17 (Neurexan; 3%) and eight (valerian; 4%) subjects in the two groups received psychotherapy. Likewise, concomitant medications for the condition were rare: four subjects (1%) in the Neurexan group and one subject (0.5%) in the valerian group were restarted on psychotropic drugs at the intermediate or the final examination.

### Therapeutic Effectiveness

In both treatment groups, symptomatic improvements were observed during the course of the study. From baseline to the end of the observation period, the scores for all study variables improved to significant degrees ($p < 0.05$). The summary score of all variables was reduced from 19.0 ± 6.1 at baseline to 7.4 ± 6.8 at the end of the observation period in the Neurexan group, a reduction of 11.5 ± 7.3 score units (Fig. 2). In the valerian group, the summary score was reduced from 21.4 ± 6.0 to 12.6 ± 7.3, a reduction of 9.0 ± 6.6 score units. The difference in least-squares mean changes between the groups from baseline to the end of the observation period was 3.9 ± 6.3 in favor of Neurexan therapy. The changes from baseline and the differences between the groups were statistically significant.

The differences in effectiveness between the groups were consistent for all study variables, with significantly greater improvements in the Neurexan group than in the valerian-based group (Fig. 3). The only exception was headache/pressure, for which there was a nonsignificant trend towards greater benefits with Neurexan (Fig. 3). All significant differences in favor of Neurexan were observed at a significance level of $p < 0.001$. The lower border of the 95% confidence interval did not cross the line of unity for any of the study variables.

In concordance with the changes in scores for individual variables, perceived global state of nervousness/restlessness improved in a majority of subjects in both groups, with a greater degree of improvement in the group receiving Neurexan therapy (Fig. 4A; $p < 0.0001$ for the comparison between treatments). One-third of the subjects (204; 35.7%) in the Neurexan group reported "very much improved" compared with 21 subjects (9.4%) receiving valerian. In the assessment of subjects' overall health status at the end of the observation treatment, 242 subjects (42.4%) reported "excellent" therapeutic effectiveness on their overall health status, compared with 24 subjects (10.7%) in the group receiving valerian-based therapy (Fig. 4B; $p < 0.0001$ for the comparison between treatments).

The time to improvement was shorter with Neurexan therapy than in the group receiving valerian therapy (Fig. 5). The differences between therapies were significant ($p < 0.01$).

### Tolerability and Subject Compliance

Both therapeutic regimens were very well tolerated, with 527 out of 577 Neurexan subjects (91.3%) and 204 out of 230 valerian subjects (88.7%) reporting "excellent" or "good" tolerability (differences between the groups were not significant). Tolerability data were missing for 47 (8.1%) and 26 (11.3%) subjects in the Neurexan and valerian groups, respectively. Compliance with the therapeutic regimens was correspondingly high: physicians reported "excellent" or "good" compliance for 492 (86.2%) Neurexan

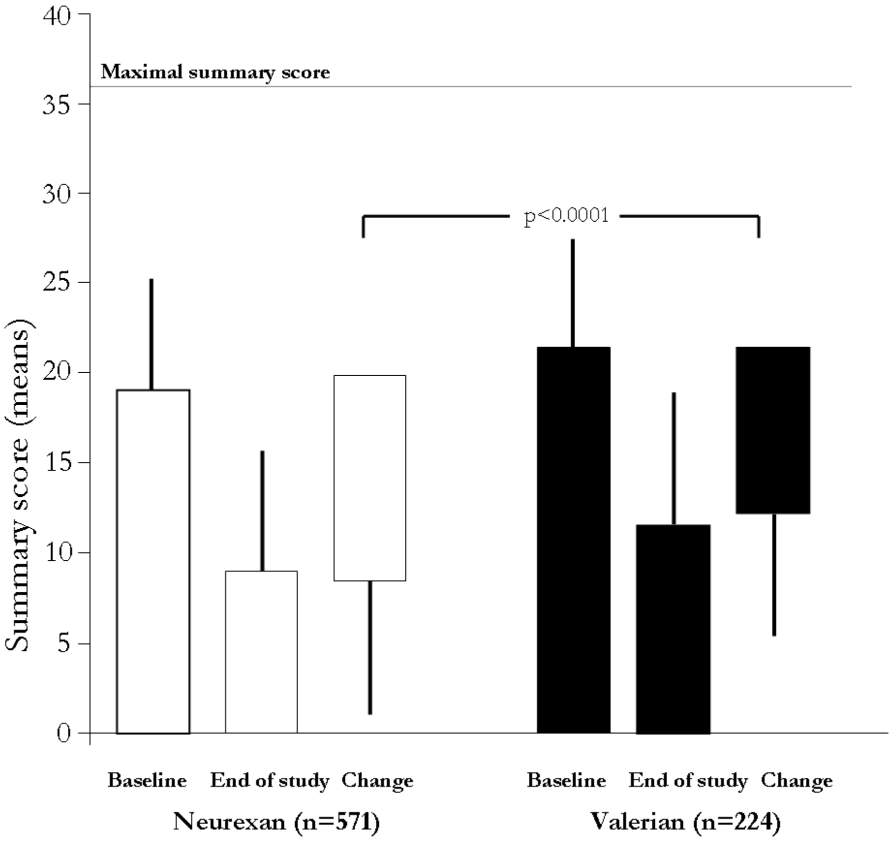

**FIGURE 2.** Summary scores for nervousness/restlessness at baseline (means) and at the end of the observation period for Neurexan (open bars) and valerian-based therapies, respectively. Also shown are mean changes from baseline to the end of the observation period. Lines indicate SD. The differences between the groups in changes from baseline in favor of Neurexan therapy were significant (ANOVA).

subjects and 178 (79.5%) valerian subjects. The difference in compliance rates between the groups was statistically significant ($p < 0.016$).

Only two adverse events were reported with possible relationship to study treatment: one case of mild nystagmus and one mild case of increased restlessness. Both occurred in the Neurexan group and none required additional medical treatment.

### DISCUSSION

The results of this large-scale observational study indicate that the homeopathic therapy Neurexan is at least as effective as valerian-based combination therapies in the symptomatic short-term treatment of nervousness/restlessness as conducted in general clinical practice. In this treatment setting, the effectiveness of Neurexan therapy was superior to that of valerian-based combination therapies on a wide range of variables, as well as on the overall summary score for nervousness/restlessness. The exception was headache/pressure, where there was a nonsignificant trend in favor of the homeopathic preparation. The superiority was also seen on the subjects' perceived overall state of nervousness/restlessness, on their overall health status, and on the time to first symptomatic improvement with therapy. Whether based on changes assessed by the physician, by the subject, or by both in a dialogue, the analyses were consistent, supporting the reliability of the findings.

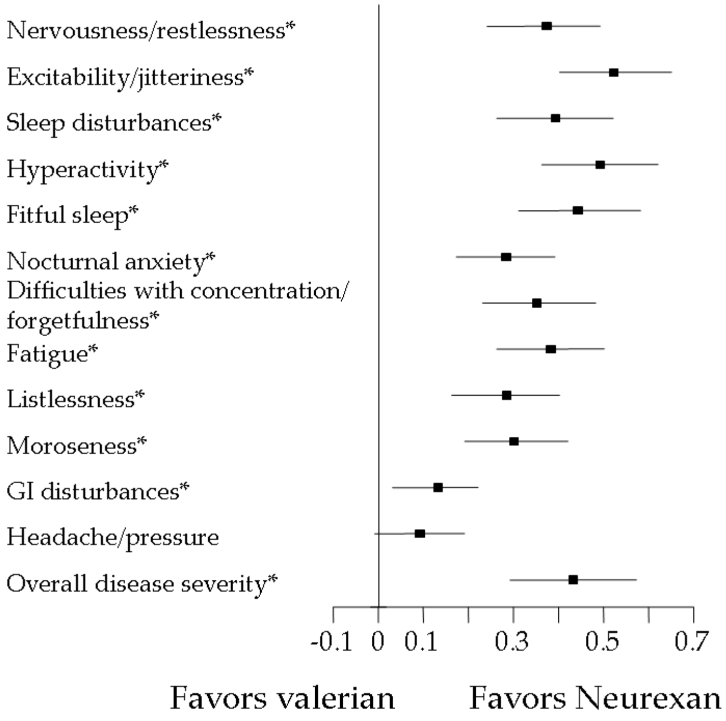

**FIGURE 3.** Point estimates (least-squares means) and 95% confidence intervals for differences between treatment groups in the overall assessment and the individual variables. Positive values indicate significantly greater benefits in the Neurexan group (n = 571); negative values indicate greater benefits with valerian-based therapies (n = 224). Asterisks indicate statistically significant differences ($p < 0.01$) in favor of Neurexan.

Nervousness and restlessness are common in the general population, although prevalence rates are hard to come by, partly because of the wide and nonstandard definition of the conditions. The same fact may lie behind the relative dearth of controlled studies addressing the effects of therapies on the condition. Difficulty in narrowing down definitions and setting clear restrictions on inclusion criteria may be a barrier to randomized controlled trials.

However, in CAM as in conventional medicine, the value and the need for basic observational research for effective clinical practice is often underestimated[23]. Without observational studies, little can be learned about prognosis and the factors that affect it, of about how effective particular therapies are under the conditions of everyday clinical practice. Factors such as relationships between practitioners and patients, in general more recognized and better developed in CAM settings, can only be studied using observational designs, sacrificing a degree of stringency to the need of relevant information[24].

Neurexan and valerian are two CAM therapies for which a substantial body of evidence is being assembled from clinical and molecular studies. The combination of valerian and lemon balm has been shown to be effective in the treatment of restlessness and dyssomnia in children[13] and in treating laboratory-induced stress in adults[14]. *Melissa* extract, as well as that of valerian, was recently shown directly to affect the primary brain enzymes responsible for gamma-aminobutyric acid (GABA) metabolism[25]. St. John's Wort has a long documented history of use in depressive and anxiety disorders[26]. *Passiflora* has been extensively studied in animal models and has been shown to be an effective adjuvant therapy in the management of opiate withdrawal[27].

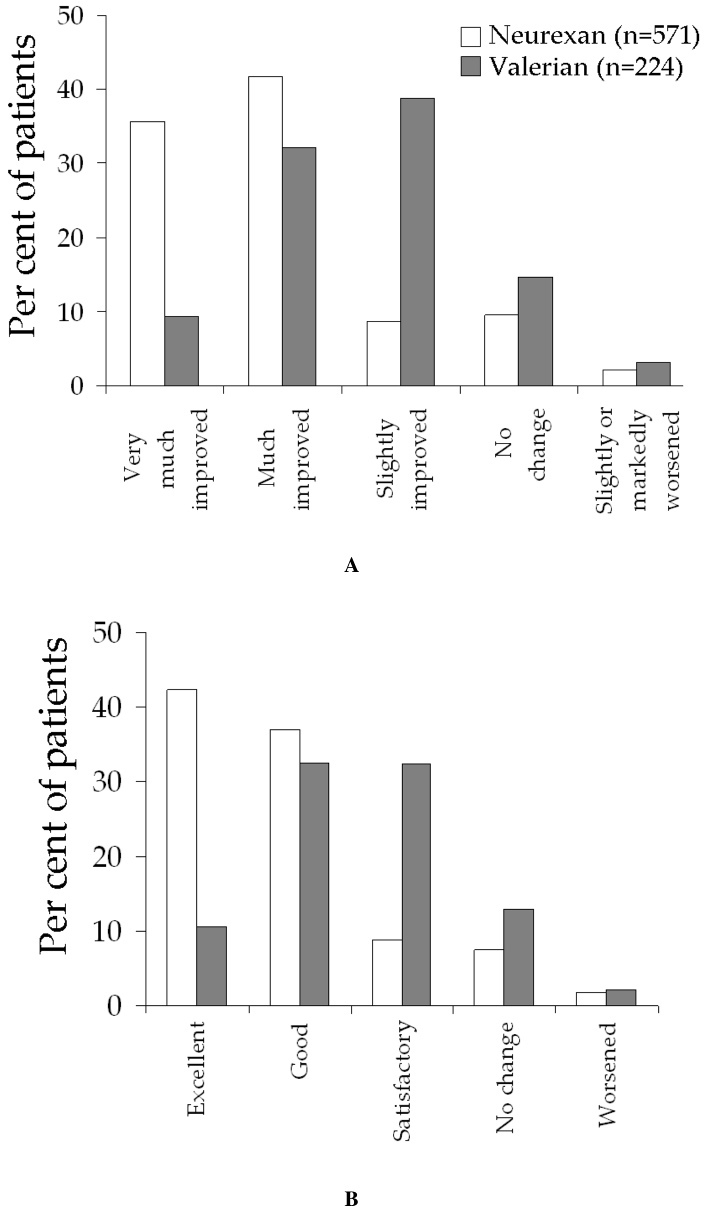

**FIGURE 4**. Effects of Neurexan and valerian-based treatments on (A) subjects' perceived global state of nervousness/restlessness and (B) subjects' overall health status. The differences in favor of Neurexan were statistically significant ($p < 0.0001$; Cochran-Mantel-Haenszel test).

In a recent observational study, Neurexan was shown to be noninferior to common valerian therapy for insomnia[8]. There are differences between this study and the present one in conditions, treatments, and results. The study of Neurexan in insomnia included half as many subjects as the current study, which reduced the power of the analysis. Whereas Waldschütz and Klein[8] focused on insomnia, the current study included sleeplessness as one variable of several, all related to the overall diagnosis of nervousness/restlessness during daytime. Thus, the results are not directly comparable.

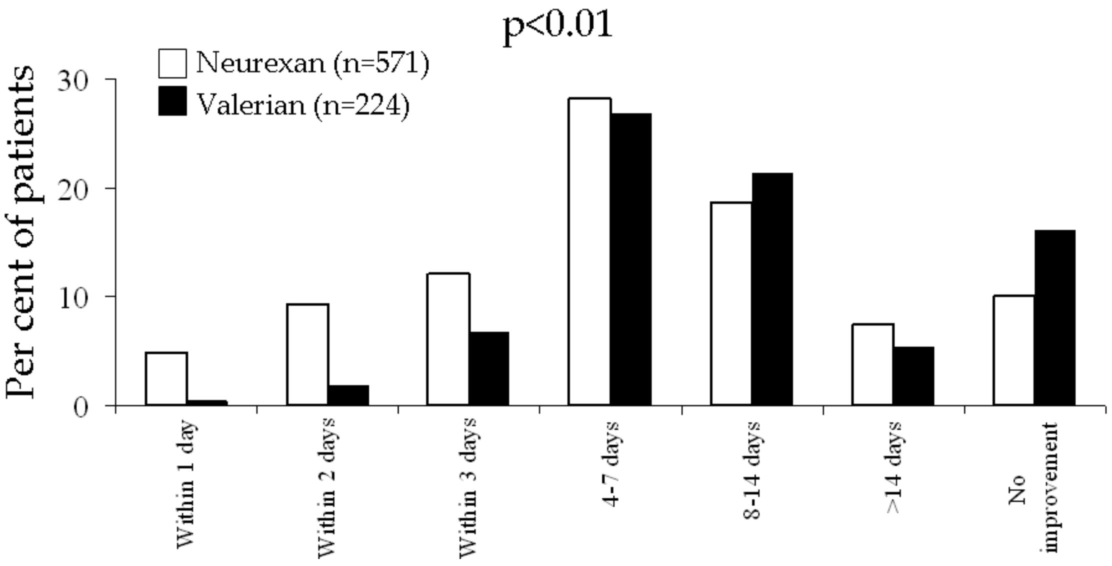

**FIGURE 5**. Percent of subjects reporting different times to first symptomatic improvement with Neurexan therapy (open bars) and valerian-based combination therapies, respectively. The differences between the treatment groups were significant ($p < 0.01$; Cochran-Mantel-Haenszel test).

The molecular basis for the effects of Neurexan remains to be elucidated in laboratory investigations. A casual look at the components list of Neurexan (Table 1) may give the misguided impression that the modes of action might be similar to those of the valerian combination therapies. This is unlikely, however. Neurexan does contain extracts of valerian (as valerianate of zinc) and *Passiflora incarnata* is one of the herbal components (together with *Avena sativa* and *Coffea arabica*). However, in accordance with the principles of homeopathy and homotoxicology, only miniscule doses of active substances are used in Neurexan to stimulate the body's own protective mechanisms[16]. In contrast, herbal medicine uses concentrations of active compounds that are several orders of magnitude greater in order to achieve direct molecular effects of a biochemical nature.

Both therapies were very well tolerated, which was to be expected based on clinical experience and published findings with Neurexan and valerian therapies.

In summary, this observational study indicates that Neurexan is an effective and well-tolerated alternative to valerian-based combination therapies for the treatment of nervousness/restlessness in subjects favorable towards a CAM-based therapy. The long-term effects of Neurexan, as well as the molecular basis for the observed benefits, would need further study.

### Limitations

There are several limitations to noninterventional studies; most importantly, the absence of a placebo group, which is unavoidable with observational study designs of effectiveness in everyday clinical practice. Thus, conclusions about the clinical efficacy of any of the treatments used cannot be delineated directly from this study type. Other potential disadvantages were the lack of blinding and randomization inherent to noninterventional studies. The wide definition of the symptoms may introduce physician and subject bias, although physicians or subjects using one kind of CAM therapy would hardly be more biased than those using another. With all nonrandomized designs, subject groups may differ at baseline, which could introduce bias and reduce the validity of the conclusions[24]. The use of propensity scores is a statistical method to reduce such effects and was used here to compensate for such differences, although differences remained for the summary score and the subscores fitful sleep, fatigue, and listlessness. It is unclear how much these differences may have contributed to the different outcomes in the respective treatment groups. However, the effects from regression to the means in a group with higher scores would be expected to be greater than in a group with lower scores. Thus, such effects would have favored subjects in the valerian group.

### ACKNOWLEDGMENTS

Financial support for the analysis was provided by Heel GmbH, the manufacturer of Neurexan. The sponsor had no influence on the conduct of the analysis.

Disclosures: R van Haselen is an employee of Heel GmbH. P Klein has received funding from Heel GmbH.
